# Bias amplification in the g-computation algorithm for time-varying treatments: a case study of industry payments and prescription of opioid products

**DOI:** 10.1186/s12874-022-01563-3

**Published:** 2022-04-25

**Authors:** Kosuke Inoue, Atsushi Goto, Naoki Kondo, Tomohiro Shinozaki

**Affiliations:** 1 Department of Social Epidemiology, Graduate School of Medicine, Kyoto University, Floor 2, Science Frontier Laboratory, Yoshida-konoe-cho, Sakyo-ku, Kyoto, 604-8146 Japan; 2 Department of Epidemiology, UCLA Fielding School of Public Health, Los Angeles, CA USA; 3 Department of Health Data Science, Graduate School of Data Science, Yokohama City University, Yokohama, Kanagawa Japan; 4 Institute for Future Initiatives, The University of Tokyo, Tokyo, Japan; 5 Department of Information and Computer Technology, Faculty of Engineering, Tokyo University of Science, Tokyo, Japan

**Keywords:** G-computation, Bias amplification, Open payments, Medicare beneficiaries, Opioids, Monte Carlo simulation

## Abstract

**Background:**

It is often challenging to determine which variables need to be included in the g-computation algorithm under the time-varying setting. Conditioning on instrumental variables (IVs) is known to introduce greater bias when there is unmeasured confounding in the point-treatment settings, and this is also true for near-IVs which are weakly associated with the outcome not through the treatment. However, it is unknown whether adjusting for (near-)IVs amplifies bias in the g-computation algorithm estimators for time-varying treatments compared to the estimators ignoring such variables. We thus aimed to compare the magnitude of bias by adjusting for (near-)IVs across their different relationships with treatments in the time-varying settings.

**Methods:**

After showing a case study of the association between the receipt of industry payments and physicians’ opioid prescribing rate in the US, we demonstrated Monte Carlo simulation to investigate the extent to which the bias due to unmeasured confounders is amplified by adjusting for (near-)IV across several g-computation algorithms.

**Results:**

In our simulation study, adjusting for a perfect IV of time-varying treatments in the g-computation algorithm increased bias due to unmeasured confounding, particularly when the IV had a strong relationship with the treatment. We also found the increase in bias even adjusting for near-IV when such variable had a very weak association with unmeasured confounders between the treatment and the outcome compared to its association with the time-varying treatments. Instead, this bias amplifying feature was not observed (i.e., bias due to unmeasured confounders decreased) by adjusting for near-IV when it had a stronger association with the unmeasured confounders (≥0.1 correlation coefficient in our multivariate normal setting).

**Conclusion:**

It would be recommended to avoid adjusting for perfect IV in the g-computation algorithm to obtain a less biased estimate of the time-varying treatment effect. On the other hand, it may be recommended to include near-IV in the algorithm unless their association with unmeasured confounders is very weak. These findings would help researchers to consider the magnitude of bias when adjusting for (near-)IVs and select variables in the g-computation algorithm for the time-varying setting when they are aware of the presence of unmeasured confounding.

**Supplementary Information:**

The online version contains supplementary material available at 10.1186/s12874-022-01563-3.

## Background

In most epidemiologic studies, there is a dilemma between including only true confounders or all possible confounders. It is often believed that including as many covariates as possible would reduce the bias due to unmeasured confounding between the treatment and outcome. However, as suggested in prior literature, this is not always true, and conditioning on variables that are associated with the outcome only through the treatment, called instrumental variables (IVs), can increase the bias of treatment effect estimates in the point-treatment settings under the presence of unmeasured confounding [1–5]. This is also the case for near-IVs, variables that are weakly associated with the outcome not through the treatment, and these covariates are often called ‘bias amplifiers’ [1, 2]. For example, a previous study demonstrated that adjusting for glaucoma diagnosis (by including it in the propensity score model)—a near-IV of statin use vs. glaucoma drugs use—moved the estimated effect of statin (vs. glaucoma drug) on mortality or hip fracture risk away from the expected effect based on the results from randomized controlled trials [6]. Some previous studies have emphasized the practical challenges to determine which variables are confounders or IVs [2, 3], suggesting the need for careful consideration of including strong predictors of the treatment in the model. If the variables are weakly associated with the outcome not through the treatment (i.e., near-IVs), it might be better to present the findings from both models with and without such variables in the point-treatment settings. However, it has been still unclear whether and the extent to which this bias amplifying feature of IVs and near-IVs can be applied to the time-varying treatment settings.

G-computation, which is the computational algorithm of g-formula, is one of the methods to estimate the causal effect of time-varying treatments accounting for time-varying confounders that are affected by the treatment [7–9]. In practice, we need the fits of 1) the regression model of the outcome on the time-varying treatments and time-varying covariates and 2) the regression models of the time-varying covariates on previous treatments and covariates, as well as the Monte Carlo integration from these model fits in the g-computation algorithm. Note that, for time-varying treatment settings, there can be IV or near-IV for each time-point which may influence the g-computation estimator in an unpredictable way through distinct regression models in the presence of unmeasured (time-fixed or time-varying) confounders. Hence, it is crucial to quantify the influence of including IV or near-IV in the regression models on the g-computation estimates.

The goal of our study is to compare the magnitude of bias due to the adjustment of IV or near-IV across different relationships between (near-)IV and treatments in the time-varying settings. After describing a case study of industry marketing payments and prescriptions of opioid products, we conducted Monte Carlo simulation studies to investigate the extent to which the bias due to unmeasured confounders is amplified by adjusting for IV or near-IV (which could also reduce bias as a proxy of an unmeasured confounder). Our study will guide readers to consider the possible magnitude of bias due to adjustment for potential IVs, and thus help them to select covariates in their g-computation algorithms under the time-varying setting in future epidemiological research.

## Methods

### A case study of industry payments and prescriptions of opioid products

Opioid overdose is a major public health crisis in the United States [10]. As initial exposure to opioid prescriptions by physicians is known as a risk factor of opioid misuse and dependence [11–13], it is important to understand the upstream determinants of physician prescription of opioids. The financial relationship between physicians and the pharmaceutical industry has received substantial attention as it may affect physicians’ clinical practice [14, 15]. Since the launch of the Open Payments program under the *Physician Payment Sunshine Act* which requires the pharmaceutical industry to publicly report data on all payments and ownership interests made to licensed physicians and teaching hospitals in 2013 [16], a growing body of literature linking the financial physician-industry relationship with physicians’ prescriptions of opioids has been published [17–21]. All previous studies consistently showed the association between the receipt (or the number of encounters) of industry marketing payments at a single time point and an increased number of opioid prescriptions. This association was observed even among physicians who already received industry payments in the previous year [21], generating a hypothesis that physicians who received the payments at multiple time points would prescribe opioids than physicians who never received the payments. However, the evidence is lacking about the potential impact of industry marketing at multiple time points on physicians’ prescription of opioid products.

In addition, while the receipt of industry payments for *non-opioids* has not been adjusted for in some previous studies, it is strongly correlated with the receipt of opioid-related industry payments [21] and may also be associated with opioid prescriptions through patient-level characteristics that are not available in the Open payments data (i.e., near-IV). For example, physicians who treat patients with severe cancer may seek an educational opportunity through industry marketing of chemotherapy, and they would also have a high chance to prescribe opioids to control pain due to cancer. This possible link poses a question of whether the results change by adjusting for the receipt of industry payments for *non-opioids*.

Therefore, we employed a g-computation algorithm to account for the time-varying confounding due to change in opioid prescription pattern over time that was partially affected by industry marketing (i.e., physicians who received industry marketing might prescribe more opioids which would motivate the pharmaceutical industry to conduct further marketing in the following year). We then compared estimated effects between models with and without the receipt of industry payments for *non-opioids*, a potential (near-)IV.

### Data sources and causal structure

We used data from the Centers for Medicare and Medicaid Services (CMS) Open Payments database 2015, 2016, and 2017 [22] linked with the CMS National Plan & Provider Enumeration System (NPPES) database [23], the CMS Physician Compare database [24], and the CMS Medicare Provider Utilization and Payment Data 2016, 2017, and 2018 [25]. We restricted physicians to those who treated Medicare beneficiaries and had physician-level characteristics, resulting in the final analytical sample of 250,944 physicians. Detailed information on each database can be found in previous literature [20, 21].

Throughout this paper, we let T_1_ and T_2_ denote the treatment at time 1 and time 2, respectively. We let Y denote the outcome of interest. We let X_1_ denote the common cause of T_1_, T_2_, and Y, let X_2_ denote the common cause of T_2_ and Y affected by T_1_, and let X_3_ denote the common cause of T_1_ and T_2_ (i.e., IV). We let Y^t1,t2^ denote the potential outcome if the treatment had taken values T_1_ = t1 and T_2_ = t2.

**Fig. 1:**
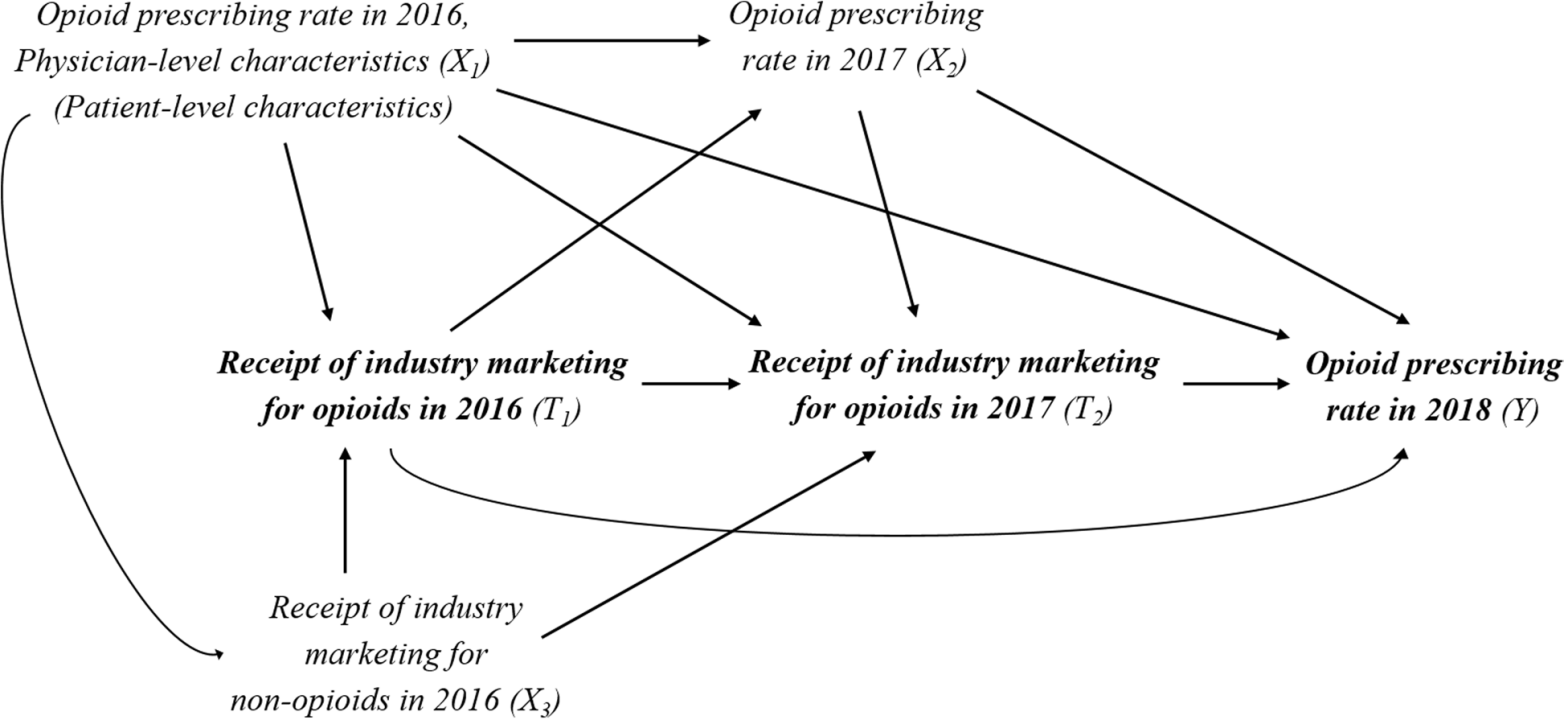
Causal diagram assumed for example about the effect of industry marketing for opioid products on physicians’ opioid prescribing rate. Patient-level characteristics were not available in the Open Payments data

Causal diagram is shown in Fig. 1. Our treatments of interest (T_1_ and T_2_) are the receipt of general payments (all forms of non-research payment including meals, speaker compensation, honoraria, travel and lodging, consulting fees, gifts, and education materials) related to opioids in 2016 (T_1_) and 2017 (T_2_). Our outcome of interest (Y) was the opioid prescribing rate (the percentage of the total claims represented by opioid claims) in 2018. Covariates at baseline (X_1_) included physicians’ sex, years in practice, specialty (30 categories listed in Supplementary Table 1), attended medical school (top-20 U.S. medical schools, U.S. medical schools ranked between 21 and 50, or others based on the *U.S. News & World Report* research ranking), the average age of beneficiaries, the proportion of male beneficiaries, average hierarchical condition category score of beneficiaries, and opioid prescribing rate in 2016. Time-varying confounder (X_2_) was the opioid prescribing rate in 2017. We considered the receipt of industry payments for *non-opioids* in 2016 (X_3_) as a near-IV that was strongly associated with X_1_ and X_2_ but was weakly associated with Y only through X_1_ and characteristics of patients (e.g., comorbidities, socioeconomic status, etc.) who each physician treated. Because such patient-level characteristics were not available in the Open Payments data while it may influence the physicians’ receipt of industry payments in general, we evaluated whether the results changed between models with and without adjustment of the receipt of industry payments for *non-opioids* in 2016. More detailed information on treatment, outcome, and covariates can also be found in previous literature [20, 21].

### Statistical analysis

We employed the following steps of the g-computation algorithm to estimate the mean difference in the opioid prescribing rate in 2018 according to the receipt of opioid-related industry marketing payments in 2016 and 2017. Analytical steps in this case study are shown as follows:

1. Fit a linear regression model to predict opioid prescribing rate in 2017 (X_2_) given the receipt of opioid-related payments in 2016 (T_1_) and baseline covariates (X_1_).
2. Fit a linear regression model to predict opioid prescribing rate in 2018 (Y) given the receipt of opioid-related payments in 2016 (T_1_) and 2017 (T_2_), baseline covariates (X_1_), and opioid prescribing rate in 2017 (X_2_).
3. Randomly assign the treatment status in 2016 (new T_1_) and 2017 (new T_2_) based on the proportion in the original dataset (i.e., new T_1_ and new T_2_ are marginally independent of all covariates [26]).
4. Predict opioid prescribing rate in 2017 (new X_2_) using the fitted regression model in step 1, newly assigned treatment status (new T_1_), and baseline covariates (X_1_).
5. Predict opioid prescribing rate in 2018 (new Y) using the fitted regression model in step 2, newly assigned treatment status (new T_1_ and new T_2_), newly predicted time-varying confounder (new X_2_), and baseline covariates (X_1_).
6. Estimate mean difference between distinct counterfactual marginal expectations of Y: E [Y^t1, t2^] – E [Y^0, 0^] for (t_1_, t_2_) = (1, 0), (0, 1), and (1, 1) where t_1_ and t_2_ were 1 when physicians received opioid-related payments in 2016 and 2017, respectively, and were 0 when they did not receive the payments in 2016 and 2017, respectively.
7. Calculate the 95% confidence interval by 200 bootstrapped samples (step 1–6).

Then, we compared the results with those when the g-computation algorithm in steps 1 and 2 additionally included the receipt of industry payments for *non-opioids* (X_3_).

### Monte Carlo simulation study

#### Causal structure and data-generating process

We conducted two Monte Carlo simulation studies to obtain quantitative results adjusting for IV or near-IV. In these simulation studies, we aimed to evaluate the difference in biases due to unmeasured confounding between models with and without adjustment of perfect IV (scenario A) or near-IV (scenario B) of the time-varying treatments. In both scenarios, we simulated 10,000 datasets of sample size (N) = 500, 10,000, and 200,000, respectively.

**Fig. 2:**
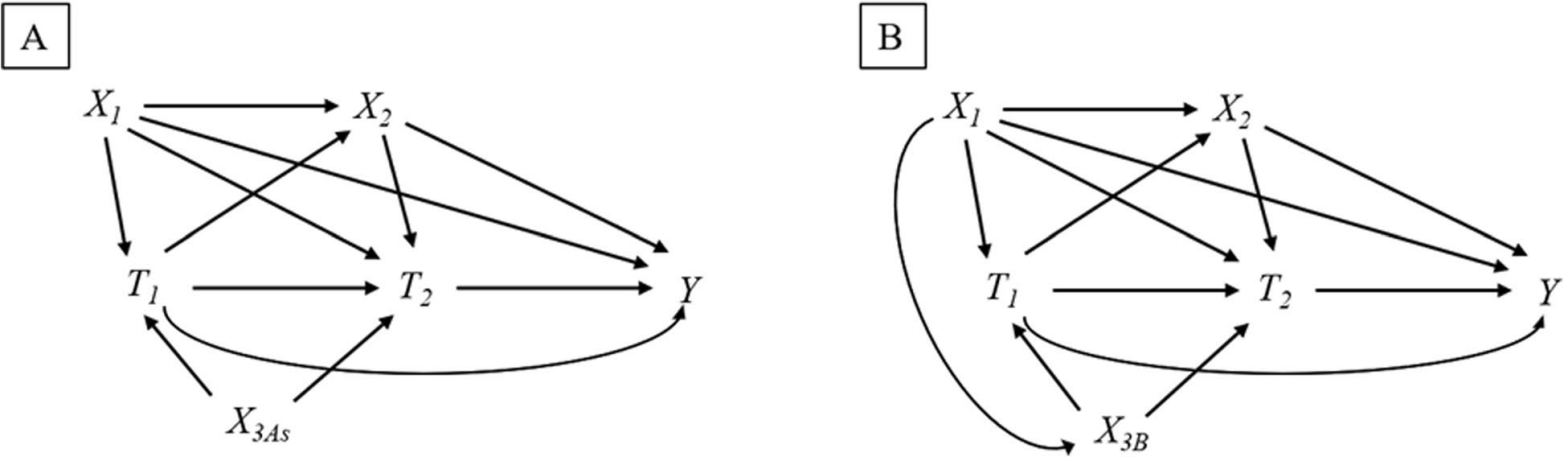
Causal diagrams for simulation studies. T_1_: treatment at time 1; T_2_: treatment at time 2; X_1_: Common cause of treatment at time 1, treatment at time 2, and outcome; X_2_: Time-varying confounder (i.e., confounder between treatment at time 2 and outcome affected by treatment at time 1); X_3_: Common cause of treatment at time 1 and treatment at time 2 (X_3As_, IV in scenario **A**; and X_3B_, near-IV in scenario **B**)

In scenario A, we assumed that X_3As_ (X_3A_1_, X_3A_2_, X_3A_3_, X_3A_4_, X_3A_5_, X_3A_6_, X_3A_7_, X_3A_8_, and X_3A_9_) are associated with Y only through T_1_ or T_2_, and therefore, perfect IVs (Fig. 2A). Each X_3As_ has a different magnitude of association with T_1_ and T_2_ as shown in Table 1. We first generated X_1_ and X_3As_ for subject *i* (=1, …, N). X_1_ was drawn from independent Bernoulli distributions with parameter 0.5. X_3As_ were drawn from independent standard normal distributions; *N (0, 1)*. Under the guidance of causal structure in Fig. 2A, we generated the status of T_1_, X_2_, T_2_, and Y for subject *i* (=1, …, N) using the following equations and values of each parameter in Table 1:

$${\displaystyle \begin{array}{c}\mathrm{logit}\left({p}_{i,t1}\right)=-10+\log (5){X}_1+{\alpha}_{3A\_1}{X}_{3A\_1}+{\alpha}_{3A\_2}{X}_{3A\_2}+{\alpha}_{3A\_3}{X}_{3A\_3}+{\alpha}_{3A\_4}{X}_{3A\_4}+{\alpha}_{3A\_5}{X}_{3A\_5}+{\alpha}_{3A\_6}{X}_{3A\_6}+{\alpha}_{3A\_7}{X}_{3A\_7}+{\alpha}_{3A\_8}{X}_{3A\_8}+{\alpha}_{3A\_9}{X}_{3A\_9}\\ {}T1i\sim \mathrm{Bernoulli}\ \left({p}_{i,t1}\right)\end{array}}$$

$${\displaystyle \begin{array}{c}\mathrm{logit}\left({p}_{i,x2}\right)=-1+\log (5){T}_{1i}+\log (5){X}_1\\ {}X{2}_i\sim \mathrm{Bernoulli}\ \left({p}_{i,x2}\right)\end{array}}$$

$${\displaystyle \begin{array}{c}\mathrm{logit}\left({p}_{i,t2}\right)=-10+\log (1.2){T}_1+\log (5){X}_1+\log (2){X}_{2i}+{\gamma}_{3A\_1}{X}_{3A\_1}+{\gamma}_{3A\_2}{X}_{3A\_2}+{\gamma}_{3A\_3}{X}_{3A\_3}+{\gamma}_{3A\_4}{X}_{3A\_4}+{\gamma}_{3A\_5}{X}_{3A\_5}+{\gamma}_{3A\_6}{X}_{3A\_6}+{\gamma}_{3A\_7}{X}_{3A\_7}+{\gamma}_{3A\_8}{X}_{3A\_8}+{\gamma}_{3A\_9}{X}_{3A\_9}\\ {}{T}_{2i}\sim \mathrm{Bernoulli}\ \left({p}_{i,t2}\right)\end{array}}$$

$${\displaystyle \begin{array}{c}\mathrm{logit}\left({p}_{i,y}\right)=-1+\log (1.2){T}_{1i}+\log (1.5){T}_{2i}+\log (1.5){T}_{1i}{T}_{2i}+\log (5){X}_1+\log (2){X}_{2i}\ \\ {}{Y}_i\sim \mathrm{Bernoulli}\ \left({p}_{i,y}\right)\end{array}}$$

**Table 1:** Assigned values of parameters in the data-generating process of scenario A

		Association with Treatment at time 2 (T_**2**_), ***γ***_***3A_s***_		
		log (2.0) i.e., OR = 2.0	log (5.0) i.e., OR = 5.0	log (10.0) i.e., OR = 10.0
**Association with Treatment at time 1 (T**_**1**_**),** ***α***_***3A_s***_	log (2.0) i.e., OR = 2.0	*X*_*3A_1*_	*X*_*3A_4*_	*X*_*3A_7*_
	log (5.0) i.e., OR = 5.0	*X*_*3A_2*_	*X*_*3A_5*_	*X*_*3A_8*_
	log (10.0) i.e., OR = 10.0	*X*_*3A_3*_	*X*_*3A_6*_	*X*_*3A_9*_

OR, odds ratio

In scenario B, we assumed that X_3B_ is associated with X_1_ in addition to T_1_ and T_2_ (Fig. 2B). In this causal structure, as X_3B_ is associated with Y through X_1_, X_3B_ is not a perfect instrument (i.e., near-IV or proxy confounder). X_1_ was drawn from independent Bernoulli distributions with parameter 0.5. Under the guidance of causal structure in Fig. 2B, we generated the status of T_1_, X_2_, T_2_, and Y for subject (*i*=1,…,N) using the following equations. To detect the possible impact of adjusting for near-IV, we assumed that X_3B_ is strongly associated with T_1_ and T_2_ (OR = 10.0) and varied its association with X_1_ as follows; *β*_1_ = 0.01, 0.05, 0.1, 0.2, and 0.3 and 0.3.

$${X}_{3 Bi}\sim N\ \left({\beta}_1{X}_1,1\right)$$

$${\displaystyle \begin{array}{c}\mathrm{logit}\left({p}_{i,t1}\right)=-6+\log (2){X}_1+\log (10){X}_{3 Bi}\\ {}T{1}_i\sim \mathrm{Bernoulli}\ \left({p}_{i,t1}\right)\end{array}}$$

$${\displaystyle \begin{array}{c}\mathrm{logit}\left({p}_{i,x2}\right)=-1+\log (5){T}_{1i}+\log (2){X}_1\\ {}X{2}_i\sim \mathrm{Bernoulli}\ \left({p}_{i,x2}\right)\end{array}}$$

$${\displaystyle \begin{array}{c}\mathrm{logit}\left({p}_{i,t2}\right)=-7+\log (1.2){T}_{1i}+\log (5){X}_1+\log (2){X}_{2i}+\log (10){X}_{3 Bi}\\ {}{T}_{2i}\sim \mathrm{Bernoulli}\ \left({p}_{i,t2}\right)\end{array}}$$

$${\displaystyle \begin{array}{c}\mathrm{logit}\left({p}_{i,y}\right)=-0.5+\log (1.2){T}_{1i}+\log (1.5){T}_{2i}+\log (1.5){T}_{1i}{T}_{2i}+\log (2){X}_{1i}+\log (2){X}_{2i}\ \\ {}{Y}_i\sim \mathrm{Bernoulli}\ \left({p}_{i,y}\right)\end{array}}$$

### Statistical analysis

Using the g-computation algorithm, we estimated mean difference between distinct counterfactual marginal expectations of Y:

$$\mathrm{E}\left[{\mathrm{Y}}^{\mathrm{t}1,\mathrm{t}2}\right]-\mathrm{E}\left[{\mathrm{Y}}^{0,0}\right]$$

for (t_1_, t_2_) = (1, 0), (0, 1), and (1, 1). We first fit two logistic regression models; 1) a model to predict X_2_ given T_1_ and an IV (one of X_3As_ in scenario A) or a near-IV (X_3B_ in scenario B) and 2) a model to predict Y given T_1_, T_2_, X_2_, and the same covariate used in the first regression model. Next, we used the regression coefficients obtained from these models to predict the values of the potential X_2_ and subsequently of the potential Y under a hypothetical intervention on T_1_ and T_2_.

True values of marginal expectations were approximately obtained in large (*N* = 10,000,000) sample generated with (Y^1, 1^, Y^1, 0^, Y^0, 1^, Y^0, 0^). We evaluated the biases of g-computation estimates across the 10,000 datasets. Then, we compared them with biases obtained in the model without adjusting for IV (in scenario A) or near-IV (in scenario B) under the presence of unmeasured confounding.

## Results

### A case study of industry payments and prescriptions of opioid products

Among 250,944 physicians included in this study, 10,826 (4.3%) physicians received opioid-related industry payments in 2016 only, 5773 (2.3%) physicians received opioid-related industry payments in 2017 only, and 12,558 (5.0%) physicians received opioid-related industry payments in both 2016 and 2017. Physicians’ demographic characteristics are shown in Supplementary Table 1. In Model 1 (without adjusting for the receipt of industry payments for *non-opioids*, X_3_), physicians who received industry payments for opioids in either 2016 or 2017 had a higher opioid prescribing rate in 2018 than physicians who did not receive the payments in 2016 and 2017 (physicians who received payments only in 2016, + 11.32% [95% CI, 9.87 to 12.78]; those who received payments only in 2017, + 7.43% [95% CI, 5.51 to 9.35]; and those who received payments in both 2016 and 2017, + 15.96% [95% CI, 15.24 to 16.69]; Table 2). We also found the association between the receipt of industry payments and the increased opioid prescribing rate in Model 2 (with adjusting for X_3_), but the estimated effect was smaller than those in Model 1 (physicians who received payments only in 2016, + 7.21% [95% CI, 3.95 to 10.47]; those who received payments only in 2017, + 3.63% [95% CI, 1.54 to 5.71]; and those who received payments in both 2016 and 2017, + 13.47% [95% CI, 12.20 to 14.73]).

**Table 2:** Estimated effect of industry marketing for opioid products on physicians’ opioid prescribing rate using g-computation model adjusting for time-varying confounders

Receipt of industry payments for opioids in 2016	Receipt of industry payments for opioids in 2017	Number of physicians	Adjusted mean difference (95% CI) in opioid prescribing rate in 2018	
			Model 1	Model 2
No	No	221,787	Ref	Ref
Yes	No	10,826	+ 11.32% (9.87 to 12.78)	+ 7.21% (3.95 to 10.47)
No	Yes	5773	7.43% (5.51 to 9.35)	+ 3.63% (1.54 to 5.71)
Yes	Yes	12,558	+ 15.96% (15.24 to 16.69)	+ 13.47% (12.20 to 14.73)

Model 1 includes physician characteristics (years in practice, sex, specialty, the medical school graduated), patients’ characteristics (average age of beneficiaries, proportion of male beneficiaries, average hierarchical condition category score of beneficiaries), and opioid prescribing rate in 2016. Model 2 included receipt of industry marketing for *non-opioids* in 2016 in addition to covariates in Model 1. Both models adjusted for time-varying confounder (i.e., opioid prescribing rate in 2017) using the g-computation algorithm. The 95% CIs were estimated by repeating the analyses on 200 bootstrapped samples

### Monte Carlo simulation study

**Fig. 3:**
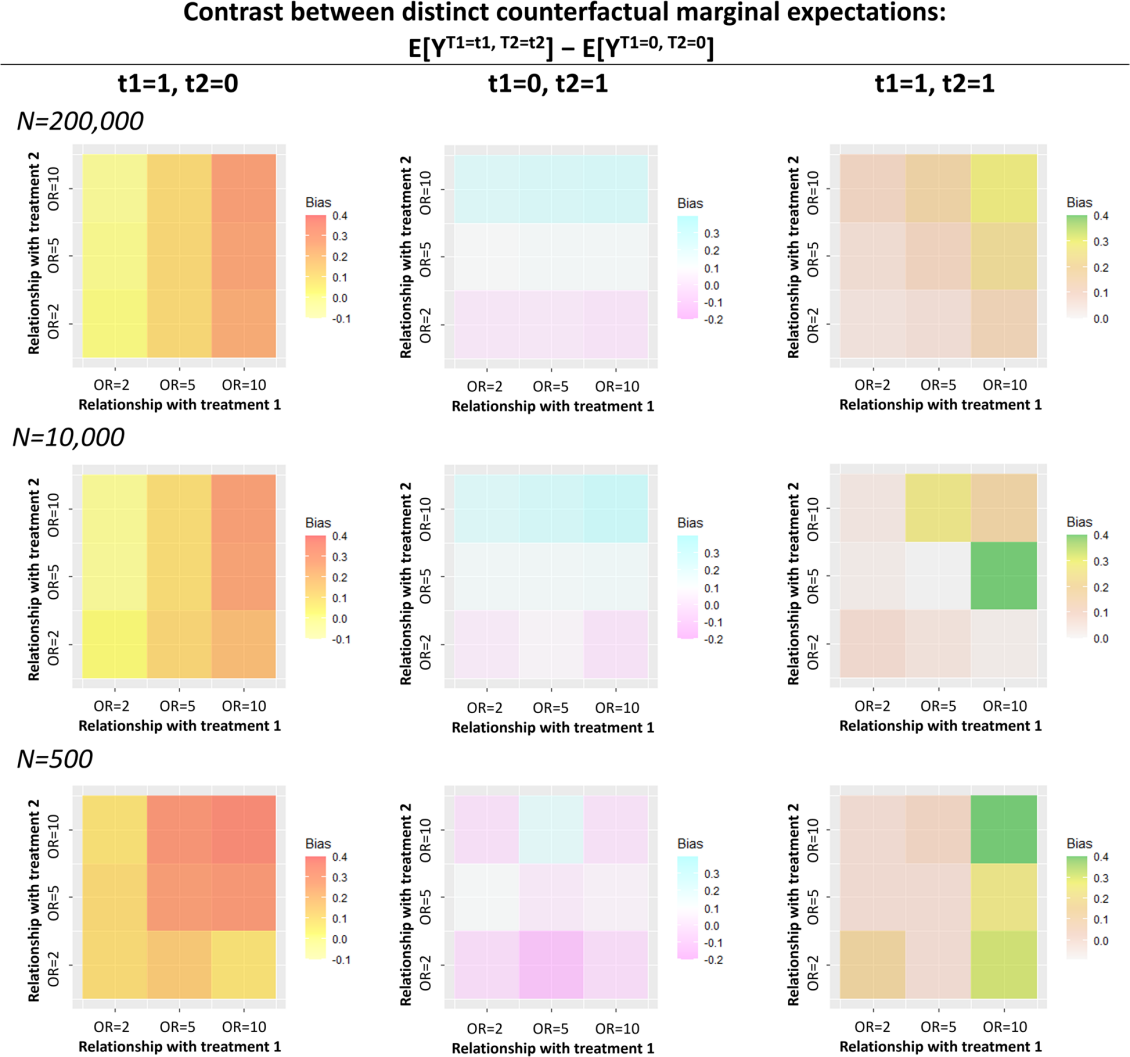
Scenario A: Increase in biases of treatment effects in the model adjusting for IV (X_3As_) compared to those in the model without adjusting for IV under the presence of unmeasured confounder (X_1_). Bias of treatment effects was calculated by subtracting true values of marginal expectations obtained in a large (*N* = 10,000,000) sample from g-computation estimates across the 10,000 datasets in each situation. Each panel shows the magnitude of increase in biases when the g-computation algorithm additionally included IV (X_3As_) which is associated with T_1_ and T_2_ (odds ratio = 2.0, 5.0, or 10.0) under the presence of an unmeasured confounder (X_1_). Biases of [Y^1, 1^] – E [Y^0, 0^] (the right column) were multiplied by − 1 to provide intuitive information on the gap from the true estimates because the true estimates of [Y^1, 1^] – E [Y^0, 0^] were negative

In scenario A, the approximate true mean difference between distinct counterfactual marginal expectation of Y under (t_1_, t_2_) = (1, 0), (0, 1), and (1, 1) were 8.4, 8.0, and 6.4 percentage point. Respectively. When we did not include IV in the model, the biases due to the presence of unmeasured confounding were 5.5 for E [Y^1, 0^] – E [Y^0, 0^], 4.3 for E [Y^0, 1^] – E [Y^0, 0^], and − 4.0 for E [Y^1, 1^] – E [Y^0, 0^]. The biases generally increased (i.e., away from the true value) when additionally adjusting for IV (Fig. 3**, **Supplementary Table 2). For example, we found the largest bias for E [Y^1, 0^] – E [Y^0, 0^] when the g-computation algorithms included IV which was strongly associated with T_1_. Likewise, we found the largest bias for E [Y^0, 1^] – E [Y^0, 0^] when the g-computation algorithms included IV which was strongly associated with T_2_. For E [Y^1, 1^] – E [Y^0, 0^], we found the largest bias when the g-computation algorithms included IV which was strongly associated with both T_1_ and T_2_. These trends were less clear when the sample size was 500 compared to when the sample size was 10,000 or 200,000. We did not find a clear trend in standard error in the models adjusting for IV in the g-computation algorithm (Supplementary Table 2).

**Fig. 4:**
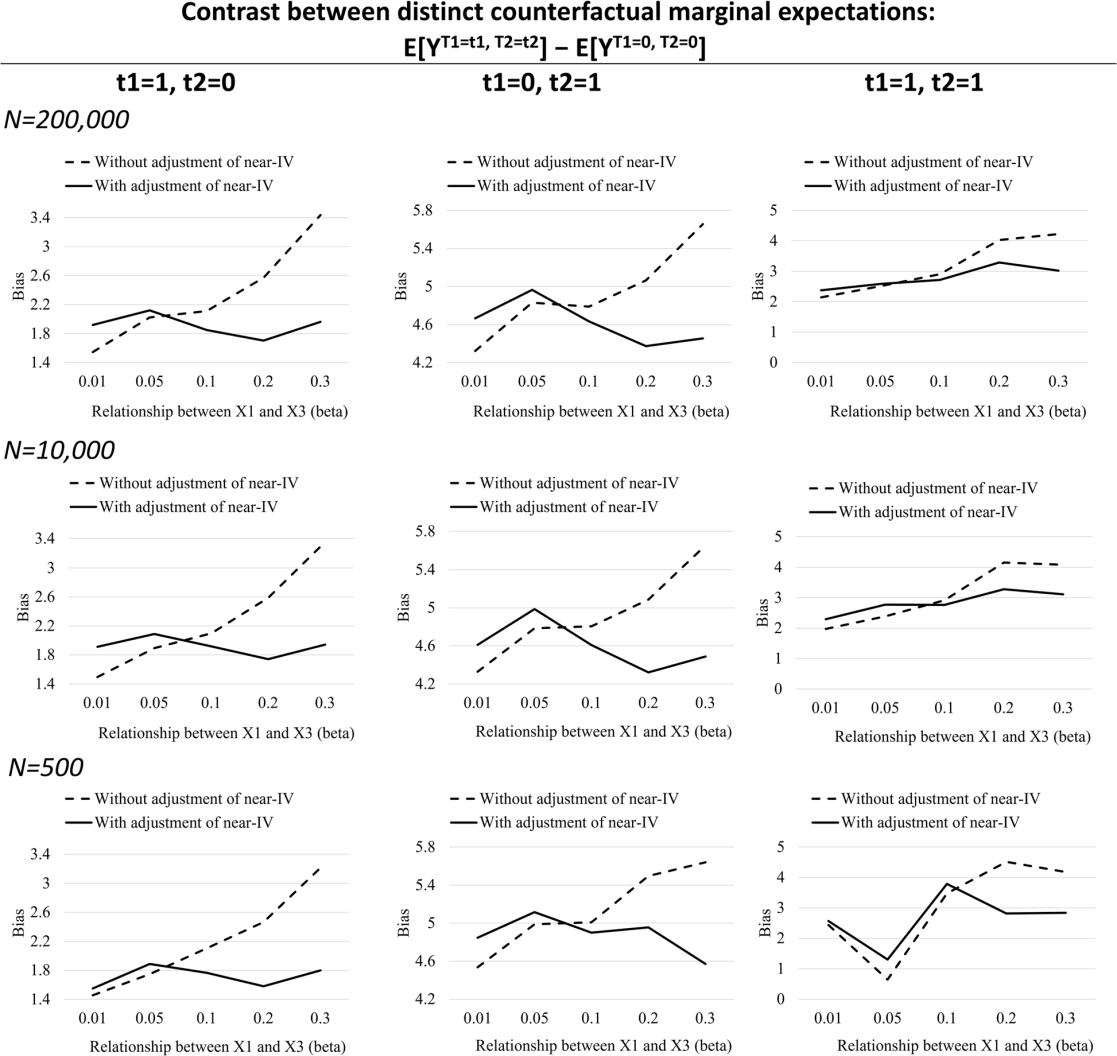
Scenario B: Comparison of bias of treatment effects between models with and without adjusting for near-IV (X_3B_) varying its relationship with unmeasured confounder (X_1_). Beta (*β*_*1*_ in *X*_*3Bi*_ ~ *N* (*β*_*1*_*X*_*1*_*, 1)*) ranged from 0.01 to 0.3. Y-axis shows bias which was calculated by subtracting true values of marginal expectations obtained in a large (*N* = 10,000,000) sample from g-computation estimates across the 10,000 datasets in each situation. Biases of [Y^1, 1^] – E [Y^0, 0^] were multiplied by − 1 to provide intuitive information on the gap from the true estimates because the true estimates of [Y^1, 1^] – E [Y^0, 0^] were negative

In scenario B, we found the bias amplification for all estimates by adjusting for near-IV (X_3B_) in the g-computation algorithm when X_3B_ had a very weak association with unmeasured confounder X_1_ (*β*_*1*_ = 0.01 or 0.05) compared with its association with T_1_ and T_2_ (OR = 10) (Fig. 4, Supplementary Table 3). However, when the association between X_3B_ and X_1_ was larger (*β*_*1*_ ≥ 0.1), we rather found the bias reduction by adjusting for X_3B_ (as a proxy of an unmeasured confounder X_1_). This trend was observed in all estimates; E [Y^1, 0^] – E [Y^0, 0^], E [Y^0, 1^] – E [Y^0, 0^], and E [Y^1, 1^] – E [Y^0, 0^].

## Discussion

Our simulation study showed that adjusting for IV of time-varying treatments in the g-computation algorithm increased bias due to unmeasured confounding, particularly when the IV has a strong relationship with the treatment of interest (i.e., T_1_ for E [Y^1, 0^] – E [Y^0, 0^], T_2_ for E [Y^0, 1^] – E [Y^0, 0^], and both T_1_ and T_2_ for E [Y^1, 1^] – E [Y^0, 0^]). Of note, we found the increase in bias even adjusting for near-IV when such variable had a very weak association with unmeasured confounders between the treatment and the outcome. Instead, the bias decreased after adjusting for near-IV when it had a stronger association with the unmeasured confounders (*β*_*1*_ ≥ 0.1, or having ≥0.1 correlation coefficient in our multivariate normal setting). Our findings were not stable with a small sample size (*N* = 500), indicating the importance of additional consideration for small sample bias.

Taken together, if there is a variable considered as a perfect IV for the time-varying exposure, we recommend not to include it in the model to obtain a less biased estimate. However, it is challenging to decide if the variable is a perfect IV or near-IV. Given that exposure is a collider between the candidate variable for IV and unmeasured exposure-outcome confounders, investigating the relationship between the candidate variable and the outcome conditional on the exposure would not overcome this challenging issue because a perfect IV can also be associated with the outcome through opened collider path due to conditioning on the exposure. Therefore, we need subject matter knowledge to determine whether the variable is a perfect IV or near-IV. If the candidate variable is likely to be near-IV for time-varying exposures, we would recommend including the variable in the g-computation algorithm because possible residual confounding would largely outweigh bias amplification due to including such a variable in the model, unless it has a very strong association with the exposure (e.g., OR > 10 with a unit standard-deviation increase) and a very weak association with the outcome (e.g., a difference of 5% standard-deviation between the presence and the absence of an unmeasured confounder). This net bias reduction is expected to be larger when the relationship between the near-IV and the unmeasured confounders gets stronger. Conducting analysis both with and without adjusting for the variable would also be an option to transparently convey the results [27, 28]. Because our simulation results were obtained through a specific data-generating process based on a case study, future studies are needed to validate our findings in other time-varying settings (e.g., different parameter values, different scales, continuous treatments, etc.).

In our case study, consistent with prior findings [17–21], we found that opioid-related industry marketing payments to physicians were associated with a higher rate of prescribing opioids in clinical practice. Moreover, using g-computation algorithms, we found that the receipt of opioid-related industry payments in 2016 was associated with opioid prescribing rate in 2018 regardless of whether they received opioid-related industry payments in 2017 or not. Although there are multiple factors that may influence opioid prescriptions such as insurance coverage, State laws, and the advent of abuse-deterrent opioid analgesics, our findings would extend the ongoing discussion about how industry marketing affects clinical practice to the time-varying setting (i.e., the possible ‘legacy effect’ of the industry payments on clinical practice) for not only opioids [17–21] but also other drugs such as cardiovascular drugs [29, 30] and insulin [31]. Our case study was based on data of licensed physicians in the US and did not include other professionals such as nurse practitioners who might have prescribed opioids during the study period. In addition, because we focused on the influence of industry marketing on overall opioid prescribing rates among physicians, whether the relationship varies across health practitioners and opioid types should be the subject of future research.

We also found that the estimated effects were generally larger in Model 1 (model without adjustment for the receipt of industry payments for *non-opioids*) than Model 2 (model with adjustment for the receipt of industry payments for *non-opioids*). If we assume there is no relationship between unmeasured confounders (e.g., patient-level characteristics, State laws, the development of abuse-deterrent opioid analgesics over the study period, etc.) and the receipt of industry payments for *non-opioids*, Model 1 would be preferred to Model 2. However, given that the association between the receipt of industry payments for *non-opioids* and the above-mentioned unmeasured confounders may not be very weak in reality, we assume that the estimated effects in Model 2 would be less biased estimates than those in Model 1 based on our simulation study.

## Conclusions

In summary, we found that adjusting for a perfect IV may amplify the bias even under the setting of time-varying treatments. This was also the case for near-IVs only when the magnitude of its association with unmeasured confounders is much weaker than that with the time-varying treatments. These findings would help researchers to consider the magnitude of bias when adjusting for (near-)IVs and select variables in the g-computation algorithm for the time-varying setting when they are aware of the presence of unmeasured confounding.

## Supplementary Information

**Additional file 1.**

## Data Availability

All data are publicly available at https://www.cms.gov.
