# Supplementary material for: Bias amplification in the g-computation algorithm for time-varying treatments: a case study of industry payments and prescription of opioid products
Source: BMC Med Res Methodol. 2022 Apr 25;22:120. doi: 10.1186/s12874-022-01563-3 (PMC9036763; doi:10.1186/s12874-022-01563-3)
Supplement: Supplementary file 1 — Additional file 1. [file 12874_2022_1563_MOESM1_ESM.pdf]

**Supplementary Table 1.** Characteristics of physicians according to receiving industry payments for opioid products in 2016 and 2017.

| Variables | Physicians who did not receive industry payments for opioids in 2016 and 2017 (N=221,787) | Physicians who received industry payments for opioids only in 2016 (N=10,826) | Physicians who received industry payments for opioids only in 2017 (N=5,773) | Physicians who received industry payments for opioids in 2016 and 2017 (N=12,558) |
|---|---|---|---|---|
| Sex, % | | | | |
| Female | 30.7 | 24.6 | 23.7 | 18.8 |
| Male | 69.3 | 75.4 | 76.3 | 81.2 |
| Years in practice, mean (SD) | 25.8 (11.7) | 27.3 (10.7) | 26.9 (11.1) | 26.7 (10.5) |
| Specialty, % | | | | |
| Primary Care | | | | |
| Family Medicine | 23.5 | 37.6 | 31.8 | 30.1 |
| Internal Medicine | 23.6 | 27.1 | 23.8 | 18.6 |
| Pediatrics | 0.8 | 0.8 | 0.4 | 0.4 |
| Hospitalist | 1.1 | 0.5 | 0.4 | 0.2 |
| Surgery | | | | |
| Orthopedic | 6.9 | 6.4 | 6.8 | 2.9 |
| General | 5.7 | 4.7 | 2.0 | 0.7 |
| Urology | 2.4 | 0.9 | 1.3 | 0.1 |
| Obstetrics/Gynecology | 1.9 | 1.0 | 0.7 | 0.4 |
| Otolaryngology | 1.6 | 0.3 | 0.3 | 0.1 |
| Neurosurgery | 0.9 | 0.7 | 0.7 | 0.4 |
| Plastic | 0.6 | 0.1 | 0.2 | 0.0 |
| Thoracic/Vascular | 0.4 | 0.1 | 0.1 | 0.0 |
| Colorectal | 0.2 | 0.4 | 0.3 | 0.1 |
| Specialists | | | | |
| Emergency Medicine | 4.1 | 1.2 | 1.1 | 1.0 |
| Psychiatry | 3.8 | 0.3 | 1.2 | 0.7 |
| Hematology/Oncology | 2.5 | 4.5 | 6.1 | 3.1 |
| Cardiology | 2.7 | 0.5 | 0.4 | 0.2 |
| Ophthalmology | 2.6 | 0.1 | 0.1 | 0.0 |
| Neurology | 1.7 | 2.0 | 6.8 | 3.8 |
| Rehabilitation | 1.2 | 2.1 | 3.7 | 11.1 |
| Anesthesiology | 0.7 | 2.2 | 4.2 | 15.0 |
| Gastroenterology | 1.4 | 0.2 | 0.2 | 0.1 |
| Rheumatology | 0.9 | 2.5 | 2.4 | 3.1 |
| Nephrology | 1.2 | 0.4 | 0.1 | 0.1 |
| Endocrinology | 1.0 | 0.2 | 0.2 | 0.1 |
| Infectious Diseases | 0.8 | 0.2 | 0.2 | 0.1 |

| | | | | |
|---|---|---|---|---|
| Allergy/Immunology | 0.8 | 0.1 | 0.2 | 0.1 |
| Pulmonology | 0.7 | 0.2 | 0.3 | 0.1 |
| Pain management | 0.2 | 0.6 | 0.9 | 4.7 |
| Others[a] | 4.2 | 2.4 | 3.3 | 3.0 |
| Medical School graduated, % | | | | |
| Ranked top 20 | 9.9 | 5.4 | 6.3 | 5.2 |
| Ranked 21-50 | 17.2 | 14.6 | 14.5 | 13.4 |
| Others | 72.9 | 80.0 | 79.2 | 81.4 |
| Average age of beneficiaries in 2016, mean (SD) | 70.3 (5.3) | 70.9 (3.9) | 70.7 (4.3) | 69.0 (5.0) |
| Proportion of male beneficiaries, %, mean (SD) | 38 (15.6) | 38.5 (11.6) | 38.4 (11.8) | 38.3 (9.3) |
| Average HCC risk score of beneficiaries in 2016, mean (SD) | 1.5 (0.6) | 1.4 (0.4) | 1.5 (0.5) | 1.5 (0.4) |
| Prescribing opioid rate in 2016, % | 11.8 (17.7) | 12.7 (17.0) | 14.3 (18.6) | 24.7 (24.6) |
| Prescribing opioid rate in 2017, % | 11.2 (17.1) | 12.0 (16.5) | 14.0 (18.5) | 24.4 (24.7) |
| Receipt of industry payments for non-opioid in 2016, % | 1.2 | 99.0 | 10.9 | 99.1 |

HCC, hierarchical condition category.

[a] Others include dermatology, oral surgery, radiology, pathology, pediatric specialty, and nuclear medicine.

**Supplementary Table 2**. Scenario B: Bias of treatment effects in the model adjusting for IV ($X_{3As}$) under the presence of unmeasured confounder ($X_1$)

| Models | Contrast between distinct counterfactual marginal expectations: $E[Y^{T1=t1, T2=t2}] - E[Y^{T1=0, T2=0}]$ | | | | | | | | | | |
|---|---|---|---|---|---|---|---|---|---|---|---|
| | t1=1, t2=0 | | | | t1=0, t2=1 | | | | t1=1, t2=1 | | |
| *N in each dataset= 200,000* | Bias | SE | RMSE | | Bias | SE | RMSE | | Bias | SE | RMSE |
| Model 1 ($X_1$ missing) | 5.63 | 0.89 | 5.70 | | 4.33 | 0.80 | 4.40 | | 4.27 | 2.25 | 4.83 |
| Model 2 (Model 1 + $X_{3A\_1}$) | 5.65 | 0.89 | 5.72 | | 4.35 | 0.79 | 4.42 | | 4.32 | 2.26 | 4.88 |
| Model 3 (Model 1 + $X_{3A\_2}$) | 5.75 | 0.89 | 5.82 | | 4.36 | 0.80 | 4.43 | | 4.34 | 2.27 | 4.90 |
| Model 4 (Model 1 + $X_{3A\_3}$) | 5.88 | 0.90 | 5.95 | | 4.34 | 0.80 | 4.41 | | 4.41 | 2.24 | 4.95 |
| Model 5 (Model 1 + $X_{3A\_4}$) | 5.63 | 0.88 | 5.70 | | 4.43 | 0.80 | 4.50 | | 4.34 | 2.24 | 4.88 |
| Model 6 (Model 1 + $X_{3A\_5}$) | 5.75 | 0.88 | 5.82 | | 4.45 | 0.80 | 4.52 | | 4.39 | 2.26 | 4.94 |
| Model 7 (Model 1 + $X_{3A\_6}$) | 5.90 | 0.88 | 5.96 | | 4.45 | 0.80 | 4.52 | | 4.50 | 2.25 | 5.03 |
| Model 8 (Model 1 + $X_{3A\_7}$) | 5.61 | 0.88 | 5.68 | | 4.55 | 0.80 | 4.62 | | 4.38 | 2.24 | 4.92 |
| Model 9 (Model 1 + $X_{3A\_8}$) | 5.75 | 0.88 | 5.81 | | 4.57 | 0.80 | 4.64 | | 4.45 | 2.28 | 5.00 |
| Model 10 (Model 1 + $X_{3A\_9}$) | 5.92 | 0.89 | 5.98 | | 4.58 | 0.80 | 4.65 | | 4.56 | 2.23 | 5.08 |
| | | | | | | | | | | | |
| *N in each dataset= 10,000* | Bias | SE | RMSE | | Bias | SE | RMSE | | Bias | SE | RMSE |
| Model 1 ($X_1$ missing) | 5.66 | 4.01 | 6.94 | | 4.32 | 3.56 | 5.59 | | 4.52 | 10.19 | 11.15 |
| Model 2 (Model 1 + $X_{3A\_1}$) | 5.69 | 4.00 | 6.95 | | 4.36 | 3.54 | 5.61 | | 4.60 | 10.41 | 11.38 |
| Model 3 (Model 1 + $X_{3A\_2}$) | 5.79 | 3.97 | 7.02 | | 4.40 | 3.51 | 5.63 | | 4.57 | 10.29 | 11.26 |
| Model 4 (Model 1 + $X_{3A\_3}$) | 5.86 | 4.00 | 7.10 | | 4.33 | 3.55 | 5.59 | | 4.54 | 10.15 | 11.12 |
| Model 5 (Model 1 + $X_{3A\_4}$) | 5.65 | 3.99 | 6.91 | | 4.45 | 3.56 | 5.70 | | 4.54 | 10.27 | 11.23 |
| Model 6 (Model 1 + $X_{3A\_5}$) | 5.77 | 4.00 | 7.02 | | 4.45 | 3.57 | 5.70 | | 4.50 | 10.20 | 11.15 |
| Model 7 (Model 1 + $X_{3A\_6}$) | 5.93 | 3.99 | 7.15 | | 4.46 | 3.55 | 5.70 | | 4.94 | 10.31 | 11.43 |

| | | | | | | | | | | | |
|---|---|---|---|---|---|---|---|---|---|---|---|
| Model 8 (Model 1 + $X_{3A\_7}$) | 5.65 | 3.97 | 6.90 | | 4.54 | 3.55 | 5.77 | | 4.56 | 10.31 | 11.27 |
| Model 9 (Model 1 + $X_{3A\_8}$) | 5.77 | 3.97 | 7.01 | | 4.58 | 3.60 | 5.82 | | 4.79 | 10.29 | 11.35 |
| Model 10 (Model 1 + $X_{3A\_9}$) | 5.95 | 4.00 | 7.17 | | 4.63 | 3.58 | 5.85 | | 4.70 | 10.18 | 11.21 |
| | | | | | | | | | | | |
| *N in each dataset= 500* | Bias | SE | RMSE | | Bias | SE | RMSE | | Bias | SE | RMSE |
| Model 1 ($X_1$ missing) | 4.98 | 18.43 | 19.09 | | 4.15 | 16.11 | 16.64 | | 4.57 | 41.54 | 41.79 |
| Model 2 (Model 1 + $X_{3A\_1}$) | 5.10 | 18.51 | 19.20 | | 4.13 | 16.11 | 16.63 | | 4.72 | 41.48 | 41.74 |
| Model 3 (Model 1 + $X_{3A\_2}$) | 5.15 | 18.27 | 18.98 | | 4.01 | 16.09 | 16.58 | | 4.26 | 41.28 | 41.50 |
| Model 4 (Model 1 + $X_{3A\_3}$) | 5.08 | 18.58 | 19.26 | | 4.13 | 16.18 | 16.70 | | 4.89 | 41.30 | 41.58 |
| Model 5 (Model 1 + $X_{3A\_4}$) | 5.10 | 18.37 | 19.07 | | 4.26 | 16.09 | 16.64 | | 3.60 | 40.64 | 40.80 |
| Model 6 (Model 1 + $X_{3A\_5}$) | 5.27 | 18.53 | 19.27 | | 4.19 | 16.18 | 16.72 | | 4.38 | 41.04 | 41.27 |
| Model 7 (Model 1 + $X_{3A\_6}$) | 5.30 | 18.53 | 19.27 | | 4.22 | 16.21 | 16.75 | | 4.82 | 40.82 | 41.11 |
| Model 8 (Model 1 + $X_{3A\_7}$) | 5.08 | 18.45 | 19.13 | | 4.15 | 16.21 | 16.73 | | 4.25 | 41.96 | 42.18 |
| Model 9 (Model 1 + $X_{3A\_8}$) | 5.31 | 18.30 | 19.06 | | 4.35 | 16.09 | 16.67 | | 4.62 | 41.12 | 41.38 |
| Model 10 (Model 1 + $X_{3A\_9}$) | 5.35 | 18.38 | 19.14 | | 4.15 | 16.15 | 16.68 | | 5.21 | 41.55 | 41.87 |

Bias was calculated by subtracting true values of marginal expectations obtained in large (N=10,000,000) sample from g-computation estimates across the 10,000 datasets in each situation. Standard error of point estimate (SE) was standard deviation of g-computation estimates in each situation. Root mean squared error (RMSE) was calculated by square root of $Bias^2$+$SE^2$.

**Supplementary Table 3**. Scenario B: Comparison of bias of treatment effects between models with and without adjusting for near-IV ($X_{3B}$) varying its relationship with unmeasured confounder ($X_1$).

| Models | Bias of the contrast between distinct counterfactual marginal expectations: $E[Y^{T1=t1, T2=t2}] - E[Y^{T1=0, T2=0}]$ | | | | | | | |
|---|---|---|---|---|---|---|---|---|
| | **t1=1, t2=0** | | | **t1=0, t2=1** | | | **t1=1, t2=1** | |
| *N in each dataset= 200,000* | Model without adjustment of near-IV | Model with adjustment of near-IV | | Model without adjustment of near-IV | Model with adjustment of near-IV | | Model without adjustment of near-IV | Model with adjustment of near-IV |
| beta = 0.01 | 1.55 | 1.92 | | 4.32 | 4.66 | | 2.14 | 2.37 |
| beta = 0.05 | 2.02 | 2.12 | | 4.83 | 4.96 | | 2.51 | 2.59 |
| beta = 0.10 | 2.11 | 1.85 | | 4.79 | 4.63 | | 2.90 | 2.71 |
| beta = 0.20 | 2.57 | 1.70 | | 5.06 | 4.37 | | 4.03 | 3.29 |
| beta = 0.30 | 3.44 | 1.96 | | 5.65 | 4.45 | | 4.22 | 3.02 |
| | | | | | | | | |
| *N in each dataset= 10,000* | Model without adjustment of near-IV | Model with adjustment of near-IV | | Model without adjustment of near-IV | Model with adjustment of near-IV | | Model without adjustment of near-IV | Model with adjustment of near-IV |
| beta = 0.01 | 1.49 | 1.91 | | 4.33 | 4.61 | | 1.98 | 2.30 |
| beta = 0.05 | 1.89 | 2.09 | | 4.78 | 4.99 | | 2.39 | 2.77 |
| beta = 0.10 | 2.10 | 1.92 | | 4.80 | 4.61 | | 2.92 | 2.77 |
| beta = 0.20 | 2.59 | 1.74 | | 5.09 | 4.32 | | 4.15 | 3.28 |
| beta = 0.30 | 3.36 | 1.94 | | 5.66 | 4.49 | | 4.08 | 3.11 |
| | | | | | | | | |
| *N in each dataset= 500* | Model without adjustment of near-IV | Model with adjustment of near-IV | | Model without adjustment of near-IV | Model with adjustment of near-IV | | Model without adjustment of near-IV | Model with adjustment of near-IV |
| beta = 0.01 | 1.46 | 1.55 | | 4.54 | 4.85 | | 2.44 | 2.57 |
| beta = 0.05 | 1.75 | 1.89 | | 4.99 | 5.11 | | 0.64 | 1.30 |
| beta = 0.10 | 2.10 | 1.77 | | 5.01 | 4.90 | | 3.48 | 3.79 |
| beta = 0.20 | 2.47 | 1.58 | | 5.50 | 4.96 | | 4.52 | 2.82 |
| beta = 0.30 | 3.21 | 1.80 | | 5.64 | 4.57 | | 4.18 | 2.84 |

Beta is the strength of the relationship between near-IV ($X_{3B}$) and unmeasured confounder ($X_1$); i.e., $\beta_1$ in $X_{3Bi} \sim N(\beta_1 X_1, 1)$.Bias was calculated by subtracting true values of marginal expectations obtained in large (N=10,000,000) sample from g-computation estimates across the 10,000 datasets in each situation.
